# Bacterial and Fungal Microbiota in *Anopheles darlingi* Exhibit Differences in Diversity Across Three Main Colombian Malaria-endemic Regions

**DOI:** 10.1007/s00248-026-02829-9

**Published:** 2026-07-10

**Authors:** Paola Muñoz-Laiton, Juan C. Hernandez-Valencia, Juan Pablo Isaza, Maisa da S Araújo, Joana Falcão Salles, Margarita M. Correa

**Affiliations:** 1 Grupo Microbiología Molecular, Escuela de Microbiología, Universidad de Antioquia, Medellín, 050010 Colombia; 2 Grupo Biología de Sistemas, Escuela de Ciencias de La Salud, Facultad de Medicina, Universidad Pontificia Bolivariana, Medellín, 050031 Colombia; 3 Plataforma de Produção e Infecção de Vetores da Malária (PIVEM), Laboratório de Entomologia, Fiocruz Rondônia, Porto Velho, RO 76812-245 Brazil; 4 Groningen Institute for Evolutionary Life Sciences (GELIFES), University of Groningen, Nijenborgh 7, P.O. Box 11103, Groningen, 9700 CC The Netherlands; 5 Universidad de Antioquia, Calle 70 No. 52-21, Medellín, 050010 Colombia

**Keywords:** Microbiota, bacteria, fungi, metabarcoding, *Anopheles darlingi*, Colombia

## Abstract

*Anopheles darlingi* is the main malaria vector in the Neotropics. Despite their importance, little is known about the microbiota composition in areas where these vectors are found. Since the microbiota may influence host biology and vector competence, it is essential to understand these microbial communities in endemic regions. This study explores the bacterial and fungal communities in *An. darlingi* from three malaria-endemic areas in Colombia and examined how geography influences microbial composition. Mosquitoes were collected from the Bajo Cauca, Pacific and Amazonas regions, and their microbiota was characterized by amplifying and sequencing the 16 S rRNA V3-V4 region for bacteria and the ITS2 region for fungi. Analysis indicated greater alpha diversity in *An. darlingi* mosquitoes from the Amazonas region. Beta diversity findings demonstrated differences in bacterial and fungal community compositions across regions, especially between Amazonas and the other two regions. Additionally, the similarity of microbial communities declined slightly with increasing geographic distance. This study significantly advances our understanding of the microbiota associated with *An. darlingi* in endemic regions of Colombia, by revealing notable differences in bacterial and fungal community composition and abundance across diverse geographical populations. Importantly, these new insights extend beyond species and geographic boundaries by providing evidence of specific fungal communities linked to the vector, paving the way for innovative microbiota-based vector control strategies in malaria vector research.

**Graphical Abstract:**

**Figure:**
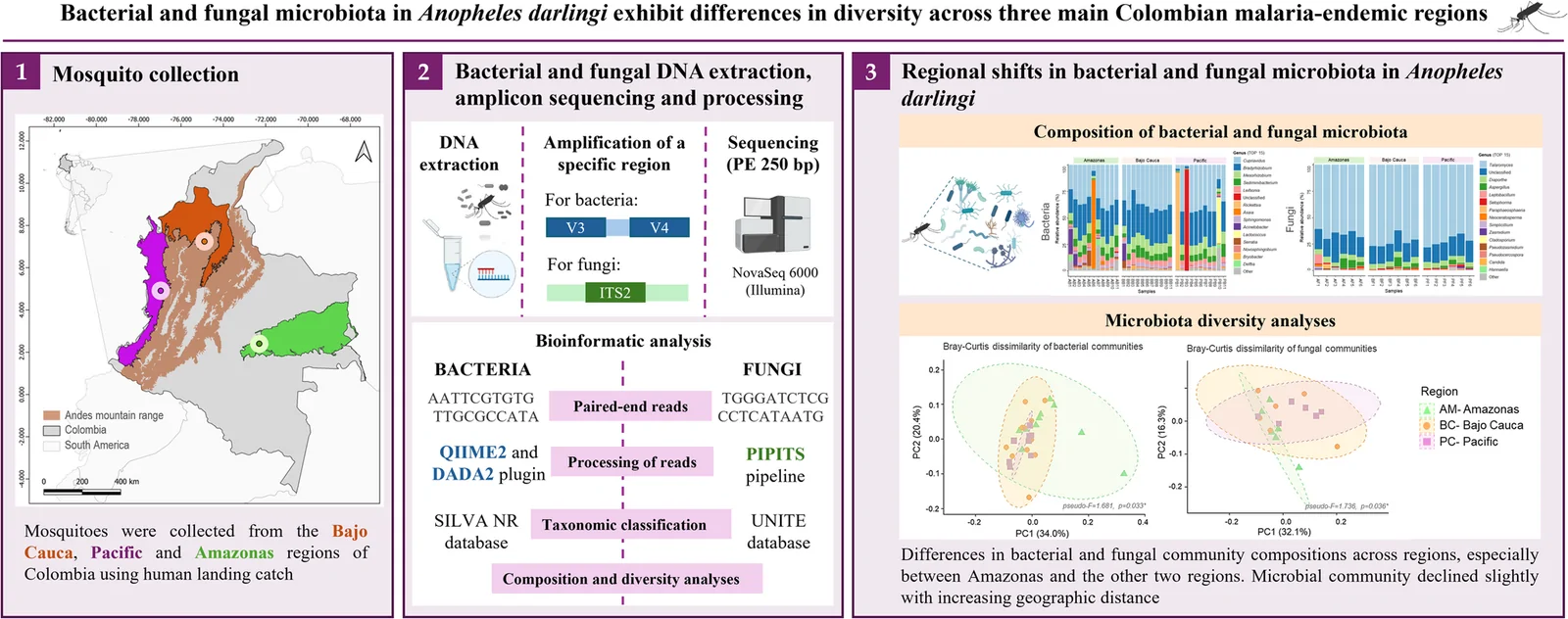

**Supplementary Information:**

The online version contains supplementary material available at https://doi.org/10.1007/s00248-026-02829-9.

## Introduction

*Anopheles* (*Nyssorhynchus*) *darlingi* Root, 1926 is the primary malaria vector in the Neotropics [1] and one of the three main vectors in Colombia [2]. It is distributed on both sides of the Andes mountain range [3], particularly in the northwest, east and Amazon regions of Colombia [4], overlapping with the primary malaria eco-epidemiological regions [2]. *Anopheles darlingi* presents plasticity in biting patterns and host blood sources [3, 5, 6], and the capacity to adapt to environmental changes [7]. Besides being a vector for *Plasmodium*, *An. darlingi* is also regarded as a holobiont, as it hosts a diverse microbiome and interacts with its associated microbial communities [8], including bacteria, fungi, viruses and protists [9].

The mosquito microbiota plays a crucial role in various aspects of vector biology, including nutrition, biological fitness and even vector competence [10]. The composition of the microbiota is variable and can be influenced by multiple factors, including mosquito species [11], developmental stage [12], host genetics [13], seasonal patterns [11, 14], and geographic location [15, 16]. However, the relative importance of these factors varies among studies. For instance, mosquito species and seasonal variation were found to be key determinants of microbiota composition [11], while in other cases, geographic location was more influential [15]. Considering the unique ecological and biological traits of each vector species, it is crucial to conduct studies tailored to specific contexts to better understand the factors influencing the mosquito microbiota in particular local environments.

The microbiota in *Anopheles* mosquitoes has been characterized mainly in African and Asian species such as *Anopheles gambiae*, *Anopheles stephensi*, *Anopheles arabiensis* and *Anopheles funestus* [17, 18]. Previous studies in *An. darlingi* from Brazil have identified the bacterial microbiota composition in the gut and salivary glands of field-captured and laboratory-reared mosquitoes [19], whereas the cultivable bacterial species associated with field-caught *An. darlingi* were identified in Colombia [20]. In addition, analysis of cultivable and non-cultivable bacteria (16 S rRNA gene sequencing) revealed that the bacterial composition of *An. darlingi* and *Anopheles nuneztovari* differed between mosquitoes from two Colombian regions, but no significant differences were observed between mosquitoes at different feeding status (blood-fed vs. unfed) or between species [15]. In contrast, the fungal component of the microbiota in mosquitoes has been less explored, even though its presence has been documented in *An. darlingi* through culture isolation followed by morphological identification and internal transcribed spacer (ITS) sequencing, in both larvae [21] and their associated breeding sites [22]. The importance of understanding the mycobiota of mosquitoes has been previously highlighted by the Mosquito Microbiome Consortium and by other experts, as ecological interactions among these components can influence host ecology, including development, survival and reproduction [23–25]. In addition, some fungal taxa have potential for vector control due to their entomopathogenic properties [26, 27]. However, based on available data, there are no reports characterizing the mycobiota in adult *An. darlingi* using a metabarcoding approach.

This study aimed to characterize the bacterial and fungal microbiota associated with adult *An. darlingi* in three Colombian malaria-endemic regions: the Amazonas, Bajo Cauca and Pacific. We hypothesize that geography shapes the bacterial and fungal microbiota of Colombian *An. darlingi*. Moreover, unlike previous studies on the microbiota of this species, this work expands the geographic sampling scope in the country by improving the taxonomic identification of the bacterial community by targeting a different 16 S rRNA gene hypervariable region (V3-V4); in addition, it constitutes the first report on the mycobiota associated with *An. darlingi* determined by ITS2 amplicon sequencing.

## Materials and Methods

### Mosquito Collection

**Fig. 1:**
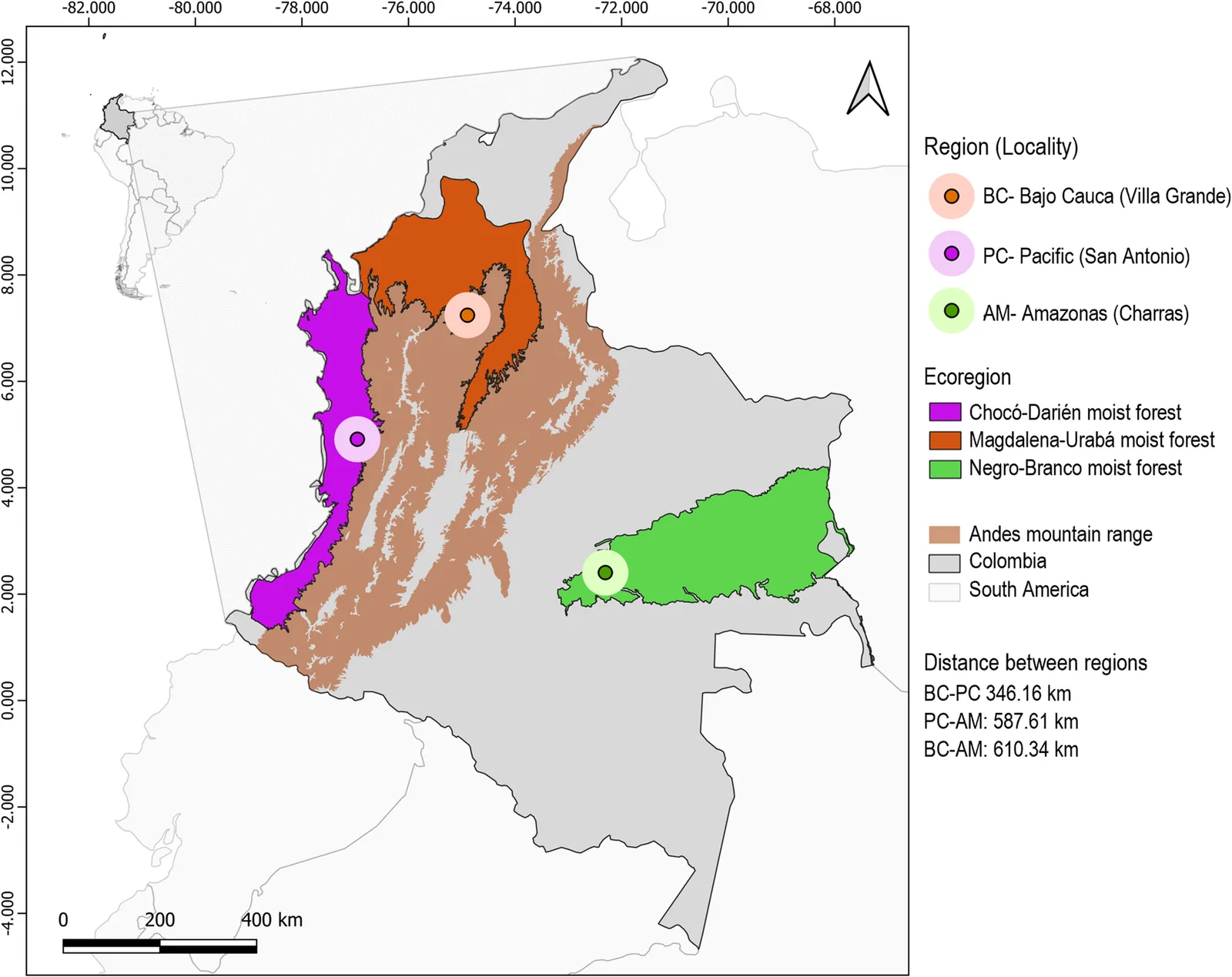
Map showing the three malaria-endemic localities and regions in Colombia where *Anopheles darling*i were collected

*Anopheles* mosquitoes were collected during 2021 from three malaria-endemic localities in Colombia: Villa Grande in the Urabá - Bajo Cauca - Sinú - San Jorge eco-epidemiological region, hereafter referred to as Bajo Cauca (BC) (7° 32’ 0,1” N; 74° 42’ 16,5” W); San Antonio in the Pacific eco-epidemiological region (PC) (5° 7’ 49,4” N, 76° 41’ 25,19” W), and Charras in the Amazonas eco-epidemiological region (AM) (02° 47’ 11,3” N, 71° 56’ 56,3” W). The Bajo Cauca region is located within the Magdalena-Urabá moist forest ecoregion, the Pacific region within the Chocó-Darién moist forest ecoregion and the Amazonas region within the Negro-Branco moist forest ecoregion [28] (Fig. 1). Mosquitoes were collected in PC, BC and AM in February, August and October, respectively, over five days at each locality. Collections were conducted between 18:00 and 00:00 h, on a single site and house, using human landing catch method both indoors and outdoors (within ~ 10 m from the house). All collections were conducted following a protocol approved by a Bioethics Committee of the University of Antioquia (CBEIH-SIU, approval code 18-35-810). To ensure study reproducibility, metadata were recorded following the recommendations of the Mosquito Microbiome Consortium [24] (Table S1). After collection, mosquitoes were anesthetized using ethyl acetate and identified to the species level using taxonomic keys [29]. Specimens identified as *An. darlingi* were preserved in DNA/RNA Shield™ (Zymo Research) and stored at -80 °C until further processing. Only unfed female mosquitoes with no apparent blood in the abdomen were included in the study according to the classification of Santos et al. (2019) [30].

### Bacterial and Fungal DNA Extraction and Amplicon Sequencing

Stored mosquito samples were thawed overnight at 4 °C and the DNA/RNA Shield solution was discarded. Each mosquito was then surface-sterilized by washing in 70% ethanol for 2–3 min, followed by three rinses with 1X phosphate-buffered saline (PBS) to remove potential external contaminants [31]. Mosquitoes were then decapitated to avoid PCR inhibition [32]. DNA was extracted from 51 individual mosquitoes (33 for bacterial and 18 for fungal analyses) using a salt precipitation protocol [33], with modifications that included an incubation step at 90 °C for 30 min after the addition of lysis buffer. Subsequently, 0.2 µL of RNase Cocktail™ Enzyme Mix (Thermo Fisher Scientific) was added, followed by a 10 min incubation at 56 °C. The extracted DNA was resuspended in 50 µL of TE buffer and stored at -20 °C until amplification. To minimize microbial contamination, all extraction reagents were filtered through sterile 0.22 μm membranes (Advantec MFS™).

DNA concentration was measured using the NanoDrop One spectrophotometer (Thermo Fisher Scientific) and the 260/280 absorbance ratio was calculated to assess purity. DNA integrity was evaluated by electrophoresis (1% agarose gel, 90 V for 40 min). Samples that met the quality criteria were amplified and sequenced by Novogene Corporation (Sacramento, CA, USA). Amplification of the bacterial 16 S rRNA V3-V4 hypervariable region was performed with primers 341 F (CCT AYG GGR BGC ASC AG) and 806R (GGA CTA CNN GGG TAT CTA AT) [34], and the fungal ITS2 region with primers ITS3-2024 F (GCA TCG ATG AAG AAC GCA GC) and ITS4-2409R (TCC TCC GCT TAT TGA TAT GC) [35]. The bacterial 16 S rRNA V3-V4 hypervariable region was selected for amplicon sequencing because it provides higher taxonomic resolution than the V1-V2 and V4-V5 regions [36] and is the most widely used marker in mosquito microbiota studies [19, 31, 37]. The fungal ITS2 region was selected because it exhibits lower length variability than ITS1 and is less prone to PCR and sequencing biases that may result in lower estimates of fungal diversity [38].

PCR amplification was performed using Phusion™ High-Fidelity PCR Master Mix (New England Biolabs) with the following thermal cycling conditions: initial denaturation at 98 °C for 1 min, followed by 30 cycles of 98 °C for 10 s, 50 °C for 30 s and 72 °C for 30 s and a final extension at 72 °C for 5 min. Library preparation was carried out using the Rapid Plus DNA Library Prep Kit (ABclonal Technology) and then 250 bp paired-end sequencing (PE250, 100k reads) was performed on the NovaSeq 6000 platform (Illumina).

To detect and remove potential external contaminants and to validate each step from DNA extraction to sequencing, two non-template controls (for bacteria and fungi), one wash control and one mock community control (for bacteria) were included following the recommendation of the Mosquito Microbiome Consortium [24]. The mock bacterial community was prepared from genomic DNA of eight bacteria previously isolated from *Anopheles* mosquitoes, with > 99% identity (V2-V4 region of the 16 S rRNA gene). The bacteria were *Acinetobacter* sp., *Chryseobacterium gleum*, *Enterobacter* sp., *Kurthia* sp., *Serratia marcescens*, *Micrococcus* sp., *Lysinibacillus* sp. and *Bacillus cereus*, obtained from the Biological Collection of Mosquitoes and Microorganisms of the Molecular Microbiology Research Group (COLBIOL-MICROMOL, 271). DNA was pooled to obtain a uniform mock community (final concentration = 104 ng/µL). Given that in Colombia there is not a fungal collection derived from *Anopheles* mosquitoes, it was not possible to assemble a fungal mock community.

### Detection of *Plasmodium* infection in *Anopheles darlingi*

All mosquito samples were screened for *Plasmodium* infection through nested PCR targeting the 18 S rRNA gene. The first amplification round was performed using primers rPLU 1 (TCA AAG ATT AAG CCA TGC AAG TGA) and rPLU 5 (CCT GTT GTT GCC TTA AAC TCC), followed by a second round with primers rPLU 3 (TTT TTA TAA GGA TAA CTA CGG AAA AGC TGT) and rPLU 4 (TAC CCG TCA TAG CCA TGT TAG GCC AAT ACC) [39, 40]. As no *Plasmodium* infected mosquitoes were detected, the infection parameter was not included in further analyses.

### Processing of Bacterial and Fungal Sequences and Taxonomic Analyses

Preprocessed bacterial and fungal paired-end reads provided by Novogene Corporation, from which primer and adapter sequences had been removed were used for downstream bioinformatic analyses. Bacterial reads were imported into QIIME2 (v. 2023.07) [41]. The DADA2 plugin [42] was used to correct sequencing errors, remove low-quality sequences and singletons, identify and remove chimeras and merge paired-end reads (parameters: trunc_len_f = 225, trunc_len_r = 222). Amplicon sequence variants (ASVs) were subsequently generated and filtered to remove non-bacterial sequences (archaeal, mitochondrial, eukaryotic and chloroplast sequences). Potential contaminants identified in non-template and wash control were detected using the isContaminant function (prevalence method) from the decontam package (v. 1.24.0) [43] in R (v. 4.4.1) [44] and were excluded from downstream analyses. Taxonomic classification was performed using the pre-formatted SILVA NR 138.1 database (v. 138.1, released 2020-08-20). Prior to taxonomic classification, 16 S rRNA V3-V4 sequences were extracted from the pre-formatted database and then the Naïve Bayes classifier [45] was trained. Extraction of reads, fitting Naïve Bayes classifier and taxonomic classification were performed using the q2-feature-classifier plugin [46].

Fungal ITS2 sequences were processed using the PIPITS pipeline (v. 3.0), specifically designed for fungal ITS data [47]. Paired-end reads were joined using the PEAR tool [48] and quality filtering was performed with FASTX-Toolkit [49]. The ITSx (v.1.1.3) [50] was used to extract the fungal ITS2 region by removing the flanking 5.8 S and 28 S rRNA sequences because these regions may complicate clustering [51, 52]. Sequences were subsequently dereplicated, singletons removed and the remaining sequences were clustered into operational taxonomic units (OTUs) at ≥ 97% identity using VSEARCH (v. 2.22.1) [53]. Chimera detection and removal were performed using the UNITE UCHIME reference database [54]. Taxonomic assignment was carried out using the RDP classifier against the UNITE database (v. 9.0) [55]. OTUs were filtered to remove non-fungal sequences, including those assigned to Metazoa, Viridiplantae and Alveolata.

### Composition, Diversity and Distance Decay Analyses of Bacterial and Fungal Communities in *Anopheles darlingi*

Alpha and beta diversity analyses were estimated with a sampling depth of 87,517 reads for bacterial and 105,044 for fungal communities. Bacterial sequences were rarefied using the core-metrics-phylogenetic plugin in QIIME2, while fungal sequences were rarefied using the rarefy_even_depth function from phyloseq (v. 1.48.0). Four bacterial sequence samples (AB6, PB1, PB3 and PB10) appeared as outliers in the principal coordinates analysis (PCoA) (95% CI); therefore, they were excluded from alpha and beta diversity analyses. However, these analyses were also performed using the full dataset, as it is known that excluding samples may lead to a loss of information (supplementary materials). Furthermore, composition analyses included all samples.

Alpha diversity indices, including Shannon’s index (H’), observed richness (S) and Pielou’s evenness (J’), were calculated to describe community diversity using the vegan package (v. 2.6.8) in R (v. 4.4.1) [44]. Normality of the data was assessed using the Shapiro-Wilk test [56]. Differences in alpha diversity were evaluated using either the Kruskal-Wallis test or ANOVA followed by pairwise comparisons, if they were significant (Dunn’s test for non-parametric data and Tukey’s HSD test for parametric data). The *p*-values were adjusted using Benjamini-Hochberg method to control the false discovery rate (FDR) (*q*-value) [57]. Beta diversity was assessed using Bray-Curtis dissimilarity and UniFrac distance matrices (both weighted and unweighted) using the phyloseq package (v. 1.48.0) in R (v. 4.4.1) [44]. Differences in mosquito microbiota composition between eco-epidemiological regions were visualized through PCoA. Statistical comparisons between groups were conducted using PERMANOVA (999 permutations). A *p*-value < 0.05 was considered statistically significant and was adjusted for multiple comparisons using FDR correction.

The relationship between the microbial composition of *An. darlingi* and geographic distance between sampling regions was assessed through a distance decay analysis. A Bray-Curtis similarity matrix (1 - Bray-Curtis dissimilarity) was constructed from the normalized abundance table of ASVs for bacteria and OTUs for fungi. Geographic distance matrices were calculated using the latitude and longitude coordinates of each sampling site and computing pairwise distances with the geosphere package (v. 1.5.20) in R (v. 4.4.1) [44]. A Spearman correlation was then performed between the matrices and the significance of the association was evaluated using the Mantel test (999 permutations) [58], in the vegan package (v. 2.6.8) in R (v. 4.4.1) [44]. Results were visualized using the ggplot2 package (v. 3.5.1).

Fungal OTUs were assigned to ecological guilds using the FUNGuild database [59]. From an initial set of 32 categories, OTUs were classified into eight ecological guilds: animal parasite/pathogen, plant parasite/pathogen, saprotroph, endophyte, epiphyte, fungal parasite, ectomycorrhizal and endosymbiont.

### Intra-kingdom Co-occurrence Network

For intra-kingdom co-occurrence/co-exclusion analysis, only normalized bacterial ASVs and fungal OTUs with a relative abundance higher than 0.1% and occurring in more than two samples were included [60, 61]. Spearman correlation analysis was applied and only correlations with a coefficient (*ρ*) > 0.7 or < -0.7 and a *p*-value < 0.05 were included. Co-occurrence networks were constructed using the Hmisc (v. 5.2.3), dplyr (v. 1.1.4) and devtools (v. 2.4.5) packages in R (v. 4.4.1) [44] and exported for visualization to Gephi (v. 0.10.1) using the Fruchterman-Reingold layout algorithm [62].

## Results

The bacterial and fungal microbiota associated with *An. darlingi* were characterized in 51 female mosquitoes; bacteria were identified in 33 specimens and fungi in 18. The sequencing of the bacterial 16 S rRNA V3-V4 region yielded 6,377,191 reads (an average of 193,248 per sample). After DADA2 filtering and removal of contaminants identified in the wash and non-template controls, 4,656,789 high-quality sequences remained, corresponding to 612 ASVs (Table S2). Fungal microbiota sequencing of the ITS2 region generated 3,045,649 reads (an average of 169,202 per sample). After processing with PIPITS, 2,917,556 sequences were retained and assigned to 1,349 fungal OTUs (Table S3). No amplification was detected in the fungal non-template control. Rarefaction curves plateaued for all samples, indicating sufficient sequencing depth (Fig. S1a, Fig. S1b).

### Bacterial and Fungal Microbiota Composition in *Anopheles darlingi* by Region

**Fig. 2:**
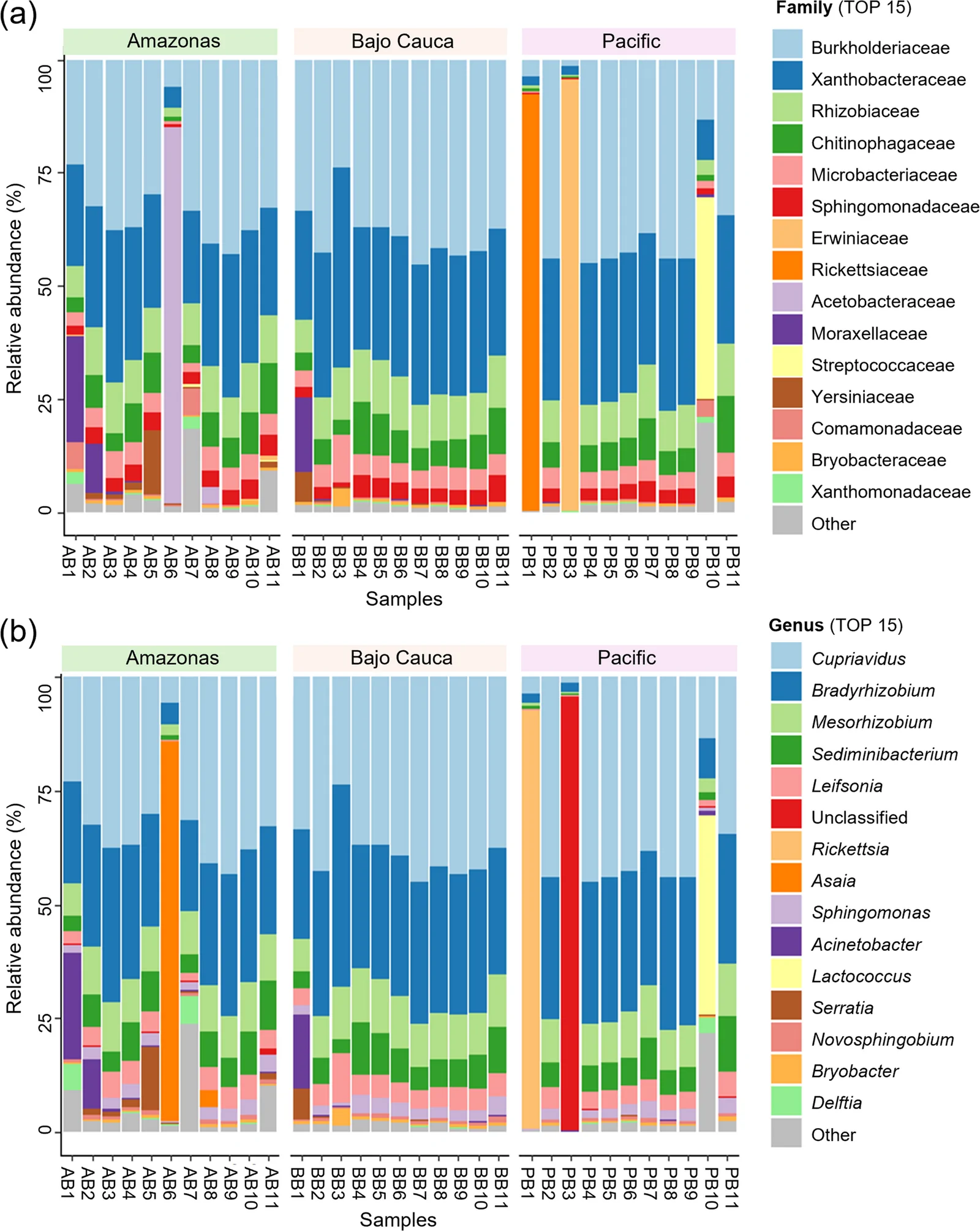
Taxonomic classification of bacterial ASVs in *Anopheles darlingi* from Colombia. **a** Family level and (**b**) genus level. The figure displays the relative abundance of the 15 most abundant taxa. Less abundant taxa were grouped under the label “Other”

Bacterial ASVs were classified into 16 phyla, 119 families and 219 genera. The most abundant phylum was Pseudomonadota (synonym: Proteobacteria), accounting for 85.3% of the total bacterial abundance, followed by Bacteroidota (6.7%), Actinomycetota (syn. Actinobacteriota, 4.9%) and Firmicutes (syn. Bacillota, 2.1%). All remaining phyla represented less than 1% of the total abundance (Fig. S2a). At the family level, the most prevalent groups were Burkholderiaceae (34.2%), Xanthobacteraceae (26.5%), Rhizobiaceae (8.9%) and Chitinophagaceae (6.1%) (Fig. 2a). At the genus level, 11 bacterial taxa accounted for approximately 90% of the relative abundance: *Cupriavidus* (33.6%), *Bradyrhizobium* (26.1%), *Mesorhizobium* (8.8%), *Sediminibacterium* (6.1%), *Leifsonia* (4.0%), *Rickettsia* (2.7%), *Asaia* (2.6%), *Sphingomonas* (2.3%), *Acinetobacter* (1.6%), *Lactococcus* (1.3%) and *Serratia* (0.8%) (Fig. 2b, Table S4). All bacteria in the mock community were identified, except for *Micrococcus*. The absence of *Micrococcus* may be due to technical issues during the preparation of the DNA mock community, potentially resulting in its underrepresentation in the sequencing dataset, although the exact cause could not be determined. It is unlikely that the cause was a sequencing error, as *Micrococcus* was successfully detected in field-collected *Anopheles* mosquitoes. 

**Fig. 3:**
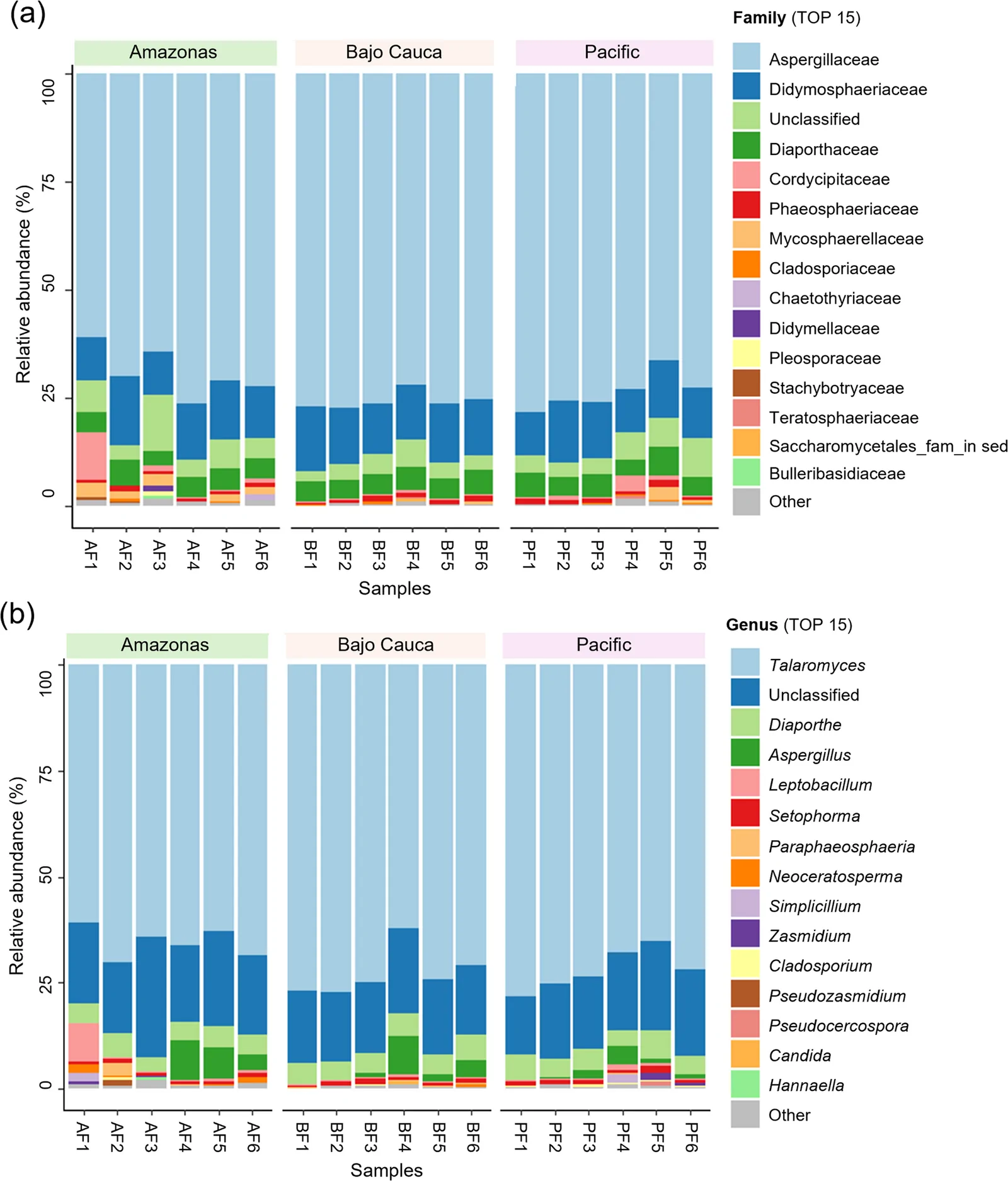
Taxonomic classification of fungal OTUs in *Anopheles darlingi* from Colombia. **a** Family level and (**b**) genus level. The figure shows the relative abundance of the 15 most abundant taxa. Less abundant taxa were grouped under the label “Other”

Fungal OTUs were classified into seven phyla, 167 families and 240 genera. The dominant phylum was Ascomycota, representing 95.4% of the total relative abundance (Fig. S2b). At the family level, the most prevalent taxa were Aspergillaceae (72.7%), Didymosphaeriaceae (12.5%), Diaporthaceae (4.8%) and Cordycipitaceae (1.2%) (Fig. 3a). At the genus level, four genera accounted for approximately 80% of the total fungal abundance, *Talaromyces* (70.0%), *Diaporthe* (4.8%), *Aspergillus* (2.5%) and *Leptobacillium* (0.9%). An additional 18.6% were not classified at the genus level (Fig. 3b, Table S5).

Among the bacterial ASVs, 133 were found in *An. darlingi* mosquitoes from all three study regions. These ASVs corresponded to 85 bacterial genera, the most abundant were *Cupriavidus*, *Bradyrhizobium*, *Mesorhizobium*, *Sediminibacterium*, *Leifsonia*, *Sphingomonas*, *Acinetobacter*, *Lactococcus* and *Serratia*. Other genera detected at lower abundances included *Streptococcus*, *Corynebacterium*, *Kurthia*, *Lysinibacillus*, *Bacillus*, *Pseudomonas*, *Aeromonas*, *Klebsiella*, *Pantoea* and *Micrococcus*, among others (Fig. S3a, Fig. S3b). Regarding the fungal component, 172 OTUs were shared across *An. darlingi* from the three regions, corresponding to 43 fungal genera. The most prominent included *Talaromyces*, *Cecropia*, *Diaporthe*, *Aspergillus*, *Leptobacillium*, *Setophoma*, *Paraphaeosphaeria*, *Zasmidium*, *Cladosporium*, *Alternaria*, *Candida*, *Trichoderma*, *Rhodotorula*, *Malassezia*, *Penicillium*, *Pichia* and *Cryptococcus* (Fig. S3c, Fig. S3d). Mosquito populations from the Amazonas region harbored the highest number of unique bacterial ASVs and fungal OTUs (115 and 878, respectively), with two to fourfold higher richness than that observed in the Bajo Cauca (38 unique bacterial ASVs, 208 unique fungal OTUs) and Pacific regions (27 unique bacterial ASVs, 445 unique fungal OTUs) (Fig. S3c, Fig. S3d). The region-specific ASVs in Amazonas were associated with bacterial genera such as *Denitratisoma* and *Lachnospira*, while unique fungal OTUs were associated with *Nigrospora*, *Trichosporon*, and *Metarhizium*, among others.

### Bacterial and Fungal Microbiota Diversity and Distance Decay Analyses in *Anopheles darlingi* by Region

**Fig. 4:**
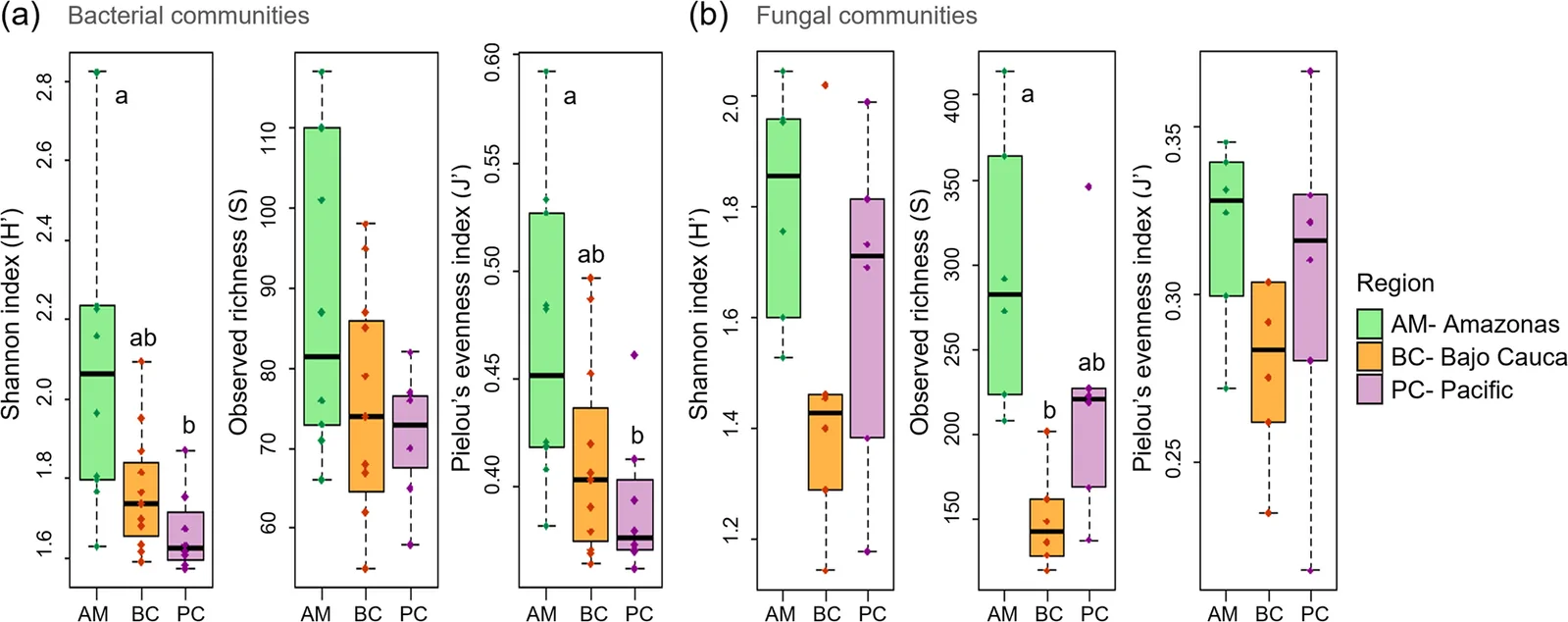
Alpha diversity of bacterial and fungal microbiota in *Anopheles darlingi* by study region. **a** Shannon diversity (Kruskal-Wallis: *p* < 0.05), observed richness (Kruskal-Wallis: *p* > 0.05) and Pielou’s evenness (Kruskal-Wallis: *p* < 0.05) for bacterial ASVs. **b** Shannon diversity (ANOVA: *p* > 0.05), observed richness (ANOVA: *p* < 0.05) and Pielou’s evenness (ANOVA: *p* > 0.05) for fungal OTUs. Different letters indicate statistically significant differences between groups (post-hoc test, *q* < 0.05)

The alpha and beta diversity of bacterial and fungal communities across different regions were assessed. The Shannon diversity index revealed significantly greater bacterial community diversity in mosquitoes from the Amazonas than in those collected in the Pacific (Dunn’s test, AM-PC, *q* = 0.002). At the same time, no significant differences were detected in the microbiota of mosquitoes from the Bajo Cauca region compared with the other regions (Fig. 4a, Table S6a). Regarding fungal communities, the Shannon index suggested higher diversity in mosquitoes from the Amazonas region, although this difference was not statistically significant (F = 2.41, *p* = 0.124). Nevertheless, observed richness indicated a significantly higher fungal richness in mosquitoes from the Amazonas compared to those from Bajo Cauca (Tukey’s HSD test, AM-BC, *q* = 0.010) (Fig. 4b, Table S6b).

**Fig. 5:**
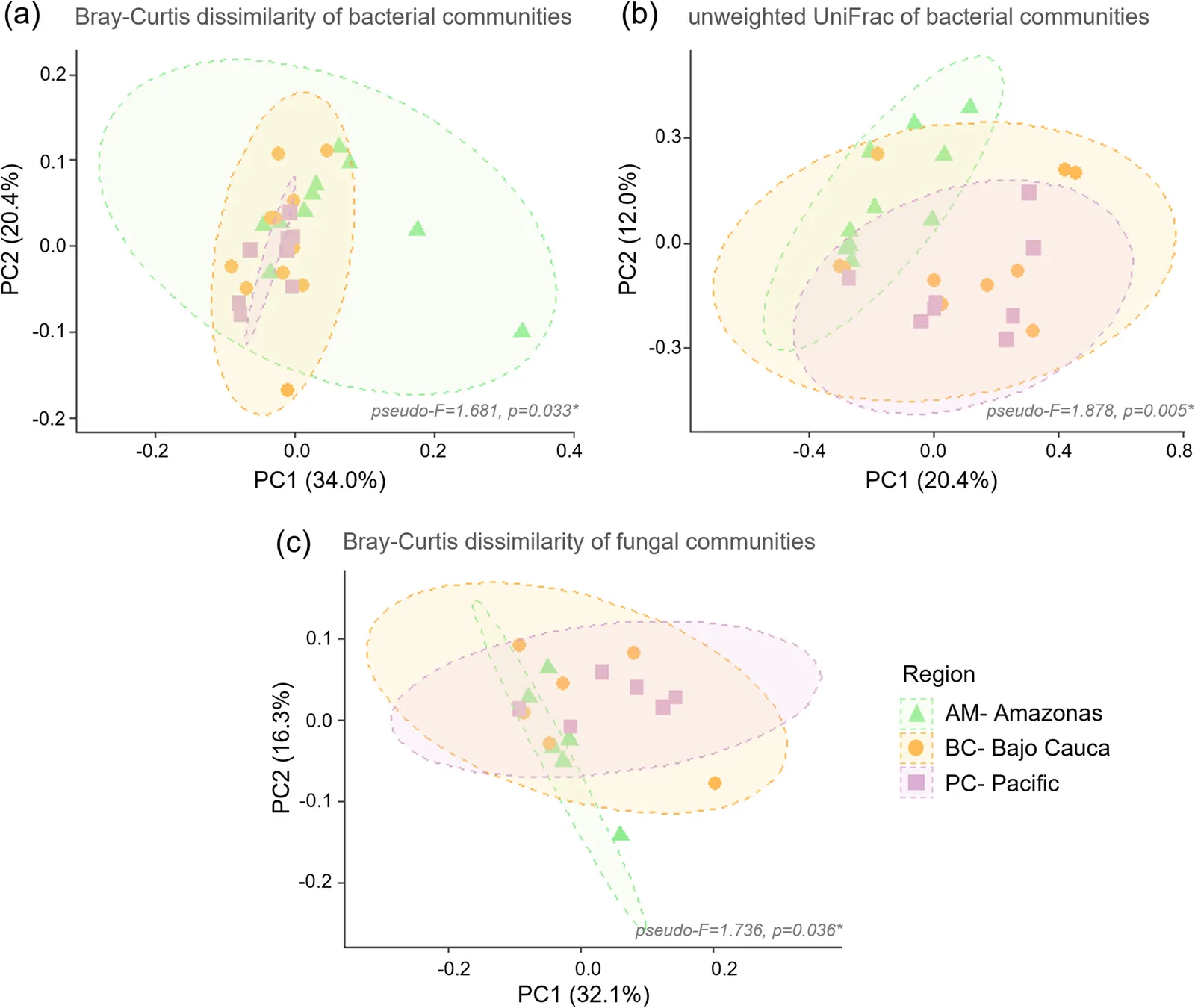
Principal coordinates analysis plots of bacterial and fungal microbiota in *Anopheles darlingi*. Based on (**a**) the Bray-Curtis dissimilarity matrix of bacterial ASVs, (**b**) the unweighted UniFrac distance matrix of bacterial ASVs, and (**c**) the Bray-Curtis dissimilarity matrix of fungal OTUs. The proportion of variation explained by each PCoA axis is indicated in parentheses. PERMANOVA statistics are shown at the bottom of each panel

Regarding beta diversity, Bray-Curtis dissimilarity analysis detected significant differences in bacterial community composition between mosquitoes from the Amazonas and Pacific regions (pairwise comparison AM-PC, *q* = 0.030) (Fig. 5a, Table S7). Unweighted UniFrac indicated differences in bacterial composition between Amazonas with both, the Pacific and Bajo Cauca regions (pairwise comparisons AM-PC, *q* = 0.003; AM-BC, *q* = 0.052, marginally significant) (Fig. 5b, Table S7). In contrast, weighted UniFrac analyses did not detect significant differences in bacterial community composition among regions (Fig. S4), and this pattern remained unchanged when all samples were included in the analysis (Fig. S5). For fungal communities, the Bray-Curtis dissimilarity analysis revealed regional microbial structure. However, pairwise comparisons between mosquitoes from Amazonas and Pacific (AM-PC, *q* = 0.055) and between Amazonas and Bajo Cauca (AM-BC, *q* = 0.055) did not reach statistical significance but were near the threshold (Fig. 5c, Table S5). Overall, results suggested that mosquitoes from the Amazonas harbored a distinct microbial structure compared to those from the Pacific and Bajo Cauca regions.

**Fig. 6:**
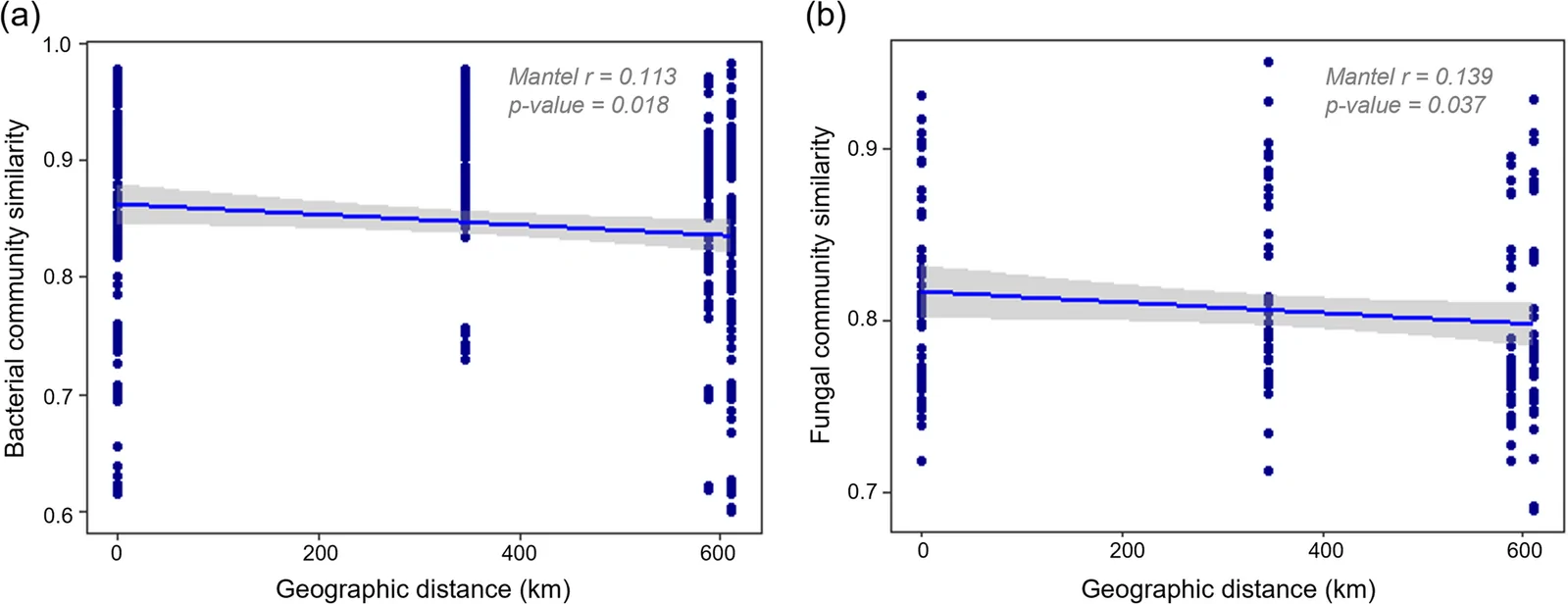
Distance decay showing Bray-Curtis similarity of the microbial community in *Anopheles darlingi* and geographic distance between regions. **a** Bacterial and (**b**) fungal communities

Results of the distance decay of microbial community similarity in *An. darlingi* revealed a low but statistically significant correlation between geographic distance and Bray-Curtis dissimilarity, for both bacterial (Mantel *r* = 0.113, *p* = 0.018) (Fig. 6a) and fungal (Mantel *r* = 0.139, *p* = 0.037) (Fig. 6b) community composition in *An. darlingi*. Increasing geographic distance was associated with decreased similarity in the bacterial and fungal communities of *An. darlingi*.

### Ecological Guilds of Fungi Associated with *Anopheles darlingi*

**Fig. 7:**
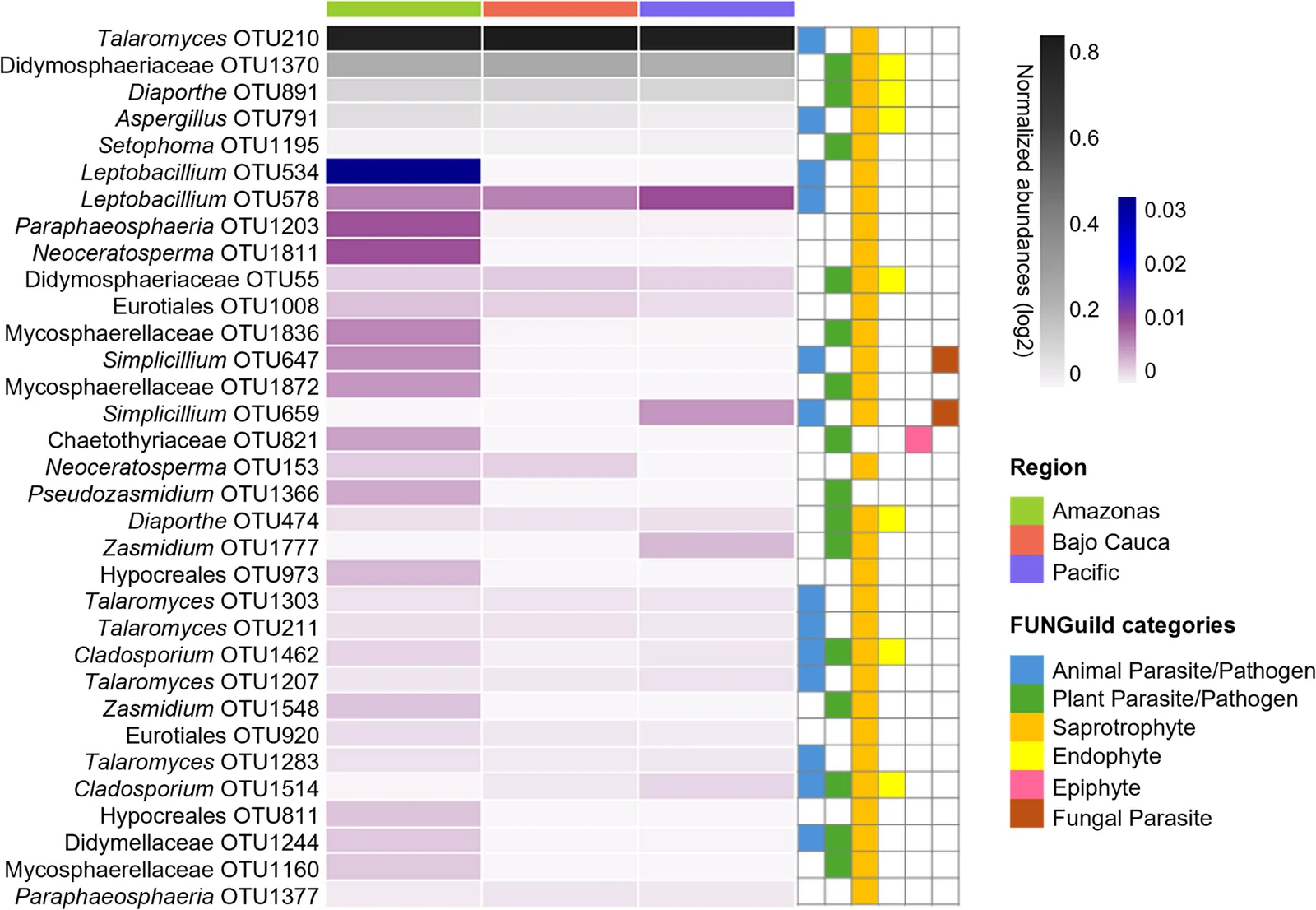
Heatmap of the 30 most abundant fungal OTUs in *Anopheles darlingi*, based on relative abundance. Each OTU was classified at the lowest available taxonomic level. The panel on the right indicates the ecological guilds inferred using FUNGuild. A secondary color scale (blue/purple tones) was applied to enhance the visualization of low-abundance OTUs

A total of 873 fungal OTUs were assigned to ecological guilds, predominantly saprotrophs (50.4%), followed by plant parasite/pathogen (17.5%), animal parasite/pathogen (14.0%) and endophytes (10.6%). Among the most abundant OTUs were: *Talaromyces*, associated with both the animal parasite/pathogen and saprotroph guilds, and *Diaporthe* and *Aspergillus*, linked to the plant parasite/pathogen, saprotroph and endophyte guilds (Fig. 7).

### Intra-kingdom Co-occurrence Network

**Fig. 8:**
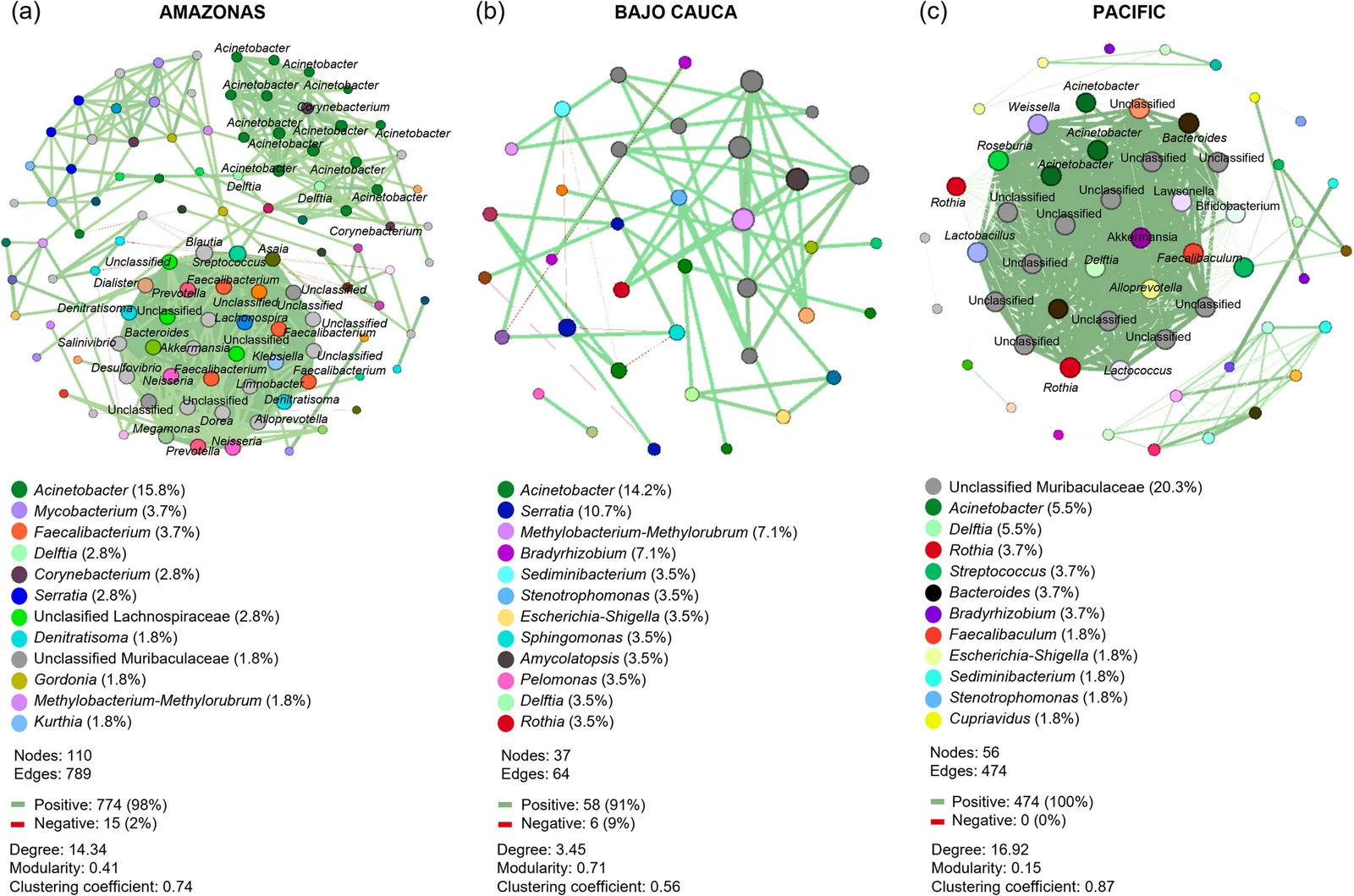
Co-occurrence networks of bacterial ASVs identified in *Anopheles darlingi* from Colombian regions. **a** Amazonas, (**b**) Bajo Cauca and (**c**) Pacific regions. The most abundant bacterial genera per region are displayed. Nodes with the same color correspond to the same genus-level taxonomic assignment. Only correlations with ρ > 0.7 or < -0.7, and *p* < 0.05 were included

In the overall bacterial co-occurrence network, ASV nodes were predominantly associated with *An. darlingi* mosquitoes from the Amazonas (Fig. S6). When networks were constructed separately by region, the bacterial network of mosquitoes from the Amazonas displayed two modules composed of highly interconnected genera or degree, including *Blautia*, *Klebsiella*, *Akkermansia*, *Prevotella*, *Bacteroides*, *Asaia* and *Acinetobacter*. Negative correlations primarily involved *Streptococcus*, which showed co-exclusion with bacteria such as *Bryobacter*, *Leifsonia*, *Lysinibacillus* and *Sphingomonas*, among others (Fig. 8a). In mosquitoes from the Pacific region, a distinct module was identified comprising *Lactobacillus*, *Delftia*, *Akkermansia*, *Weissella* and *Bacteroides* (Fig. 8c). In contrast, not highly connected ASVs were detected in mosquitoes from the Bajo Cauca region (Fig. 8b). Network stability metrics, clustering coefficient and degree were higher in the Pacific and Amazonas regions compared to the Bajo Cauca.

**Fig. 9:**
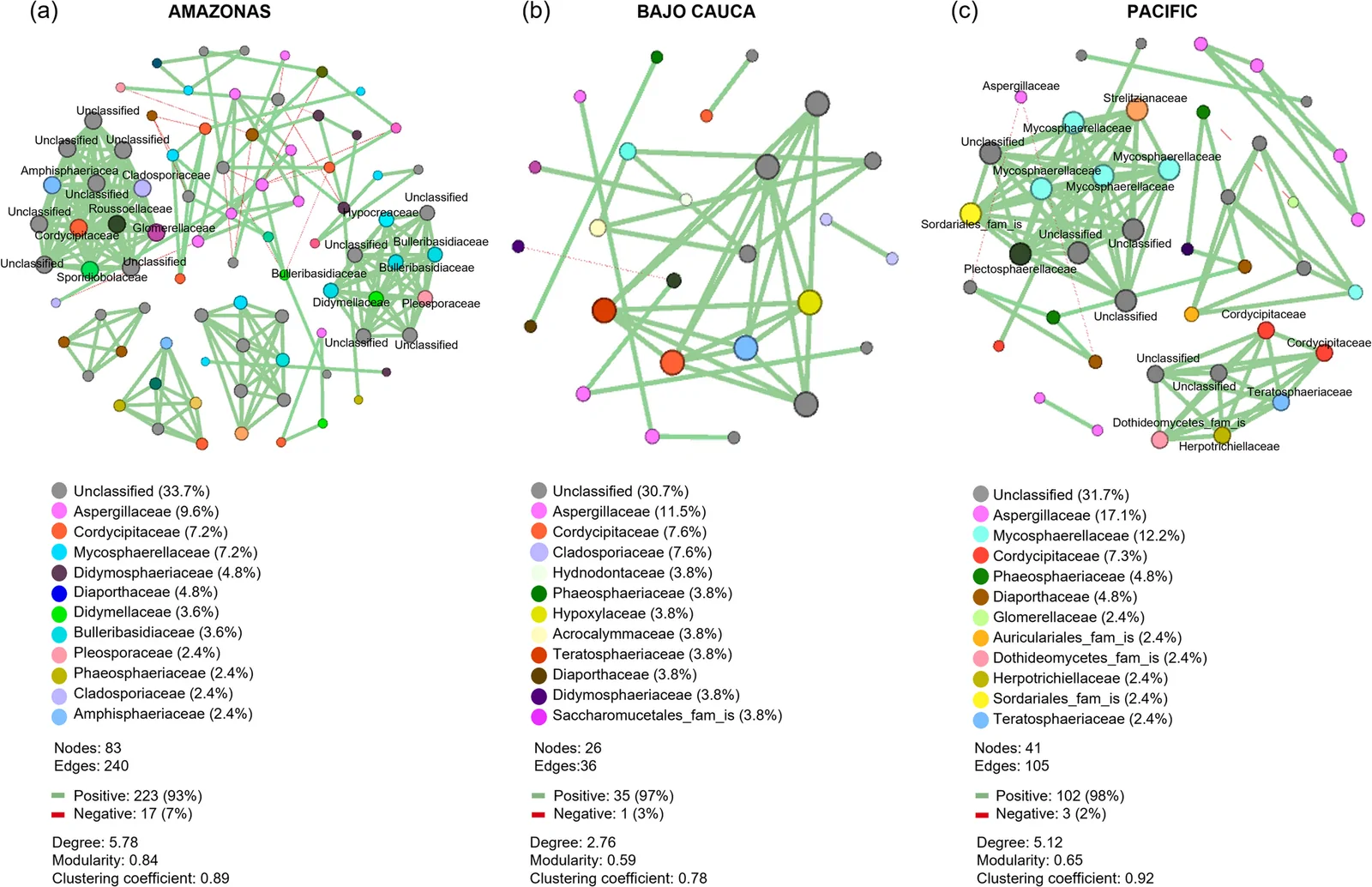
Co-occurrence networks of fungal OTUs identified in *Anopheles darlingi* from Colombian regions. **a** Amazonas, (**b**) Bajo Cauca and (**c**) Pacific regions. The most abundant fungal families per region are shown. Node colors indicate taxonomic assignment at the genus level. Only correlations with ρ > 0.7 or < -0.7 and *p* < 0.05 were included

Fungal co-occurrence networks were constructed at the family level due to a high proportion of fungal genera being designated as “Unclassified” in the intra-kingdom network. Compared with bacterial networks, the fungal networks showed a lower connectivity degree. Additionally, a higher number of OTUs were observed in *An. darlingi* mosquitoes from the Amazonas (Fig. S6b). The network of this region also exhibited the highest modularity, with more interconnected families such as *Roussoellaceae*, *Glomerellaceae*, *Sporidiobolaceae*, *Cordycipitaceae* and *Cladosporiaceae* (Fig. 9a). As seen in the bacterial networks, the Bajo Cauca region did not display a high module formation (Fig. 9b). In the Pacific region, families such as *Mycosphaerellaceae* and *Plectosphaerellaceae*, among others, were identified in the main module (Fig. 9c).

## Discussion

In this study, the bacterial and fungal communities associated with adult *An. darlingi* mosquitoes from the Amazonas, Bajo Cauca and Pacific regions of Colombia were characterized. The results revealed significant differences in microbial composition among regions, as well as a weak but significant correlation between geographic distance and microbiota composition, suggesting that geography shapes microbial composition in *An. darlingi*; in addition, environmental or biological factors likely contribute to the observed variation. This study expands the geographic scope of previous studies on bacterial communities in this species in Colombia and represents the first metabarcoding-based characterization of the fungal component in adult *An. darlingi* for the Neotropics.

Although a previous study characterized both the bacterial and fungal microbiota of this vector [63], it employed a metatranscriptomic approach designed to identify the transcriptionally active microbiota and its functional profile through RNA-Seq. In contrast, the present study is based on DNA metabarcoding, enabling the characterization of both bacterial and fungal communities, including microorganisms that are not transcriptionally active and may constitute a microbial reservoir capable of responding to environmental changes [64]. Furthermore, as the same molecular markers and analytical framework can be applied across samples, the metabarcoding approach allows robust comparisons of microbial richness, diversity and geographic variation in community composition. Together, metatranscriptomic and metabarcoding approaches provide complementary perspectives, contributing to a more comprehensive understanding of microbiota–vector interactions and their ecological dynamics.

### Bacterial Taxonomic Composition Across Regions and its Potential Role in Host Ecology

In general, the bacterial composition of *An. darlingi* included genera such as *Cupriavidus*, *Bradyrhizobium*, *Sphingomonas*, *Acinetobacter*, *Lactococcus*, *Serratia*, *Chryseobacterium*, *Lysinibacillus*, *Enterobacter*, *Streptococcus*, *Delftia*, *Escherichia-Shigella*, *Pseudomonas*, *Bacillus*, *Aeromonas*, *Pantoea*, *Micrococcus*, among others. Consistent with the present study, previous reports have identified several of these genera in *An. darlingi* from Colombia, including *Aeromonas*, *Acinetobacter*, *Pseudomonas* [15, 20], *Pantoea*, *Micrococcus*, *Bacillus*, *Serratia* and *Chryseobacterium* [20]. Similarly, adult *An. darlingi* mosquitoes collected in Brazil have been shown to harbor *Streptococcus*, *Corynebacterium*, *Acinetobacter*, *Pseudomonas*, *Staphylococcus*, *Bacillus*, *Delftia*, *Escherichia-Shigella*, *Enterobacter*, *Serratia*, *Pantoea* and *Cupriavidus* [19]. In addition, studies on *An. darlingi* feces reported the presence of *Enterobacter*, *Serratia*, *Acinetobacter*, *Staphylococcus and Pantoea* [65]; while, in breeding-site water samples, the genera found were *Acinetobacter*, *Bacillus*, *Escherichia-Shigella* and *Staphylococcus*, among others [66]. All these genera were also detected in the *An. darlingi* populations from the three Colombian regions included in this study. In addition, a subset of bacterial taxa was consistently detected in *An. darlingi* across the three Colombian regions; they most likely represent members of the vector core microbiota. This hypothesis is supported by their widespread geographic distribution in *An. darlingi* populations across the country and by their detection in *Anopheles* mosquitoes collected in Colombia in 2015 [15, 20], suggesting temporal persistence in this vector, although longitudinal studies are needed to confirm temporal stability.

Among the bacteria identified and shared across wild populations of *An. darlingi*, some genera have been reported with the potential to inhibit *Plasmodium* parasites; these include *Serratia* [67, 68], *Enterobacter* [69], *Delftia* [70], *Pantoea* [68], *Pseudomonas* [68, 71] and *Escherichia* [71]. In addition, members of the genus *Acinetobacter* sp., which has also been reported in various *Anopheles* species from Asia [72, 73] and Africa [74], demonstrated in vitro capability to inhibit *Plasmodium* development [68]. Furthermore, some species of this genus, such as *Acinetobacter baumannii* and *Acinetobacter johnsonii*, isolated from mosquitoes, have demonstrated their role in the degradation of substrates such as α-ketovaleric acid and glycine (components of blood), as well as 4-hydroxybenzoic acid and xylose (found in plants). The above supports the hypothesis that some bacteria may function as symbionts involved in blood and nectar digestion in mosquitoes [75]. Although in this study species-level classification was not possible, a recent study by our research group reported that *Acinetobacter* strains isolated from the midgut of *An. nuneztovari* and *An. darlingi* present a high 16 S rRNA gene similarity with *A. baumannii* [20].

The genera *Cupriavidus*, *Bradyrhizobium* and *Mesorhizobium* were found in greater abundance, they have previously been described as soil-associated taxa [76–78]; however, previous studies have also reported the presence of *Cupriavidus* in *Aedes* mosquitoes [79], while *Mesorhizobium* has been detected in *Aedes*, *Culex* [80] and *Anopheles* species; nevertheless, the mechanisms by which these bacteria are acquired within mosquito populations remain poorly understood [81]. Notably, some species of *Cupriavidus*, such as *Cupriavidus metallidurans*, are capable of surviving in environments contaminated with heavy metals, including mining sites [82]. In Colombia, *Anopheles* larvae have been reported in breeding habitats derived from human activities such as mining [83], and adult *Anopheles* mosquitoes have been documented in areas disturbed by human activities, including open-pit mining [84, 85]. These observations suggest that *Cupriavidus* may be acquired from the larval breeding habitats and persists in the adult stage. Furthermore, the genus *Bradyrhizobium* is associated with soils and leguminous roots owing to its nitrogen-fixing capacity [76]. Yet, a previous study reported that *Bradyrhizobium* strains isolated from ants form a distinct clade from the nitrogen-fixing rhizobial symbionts commonly associated with legume [86], suggesting that these bacteria may represent a specialized evolutionary lineage associated with insects. In line with this hypothesis, some insects are thought to depend on nitrogen-fixing bacteria [87]. Although its relationship with mosquitoes remains largely unexplored, *Bradyrhizobium* has previously been identified as part of the transcriptionally active microbiota of *An. darlingi* [63]. Taken together, these observations suggest that the ecological roles of these bacteria, as well as their interactions with *An. darlingi*, warrant further investigation.

Notably, in mosquitoes from the Pacific region, there was a high abundance of *Rickettsia* in sample PB1 (> 90% relative abundance) and a lower abundance in sample PB5. *Rickettsia* species are strictly obligate intracellular bacteria, some species are pathogenic to mammals [88]. The presence of this bacterium has been previously reported in mosquitoes of the genera *Anopheles* [89], *Aedes* and *Culex* [90]. Although the role of mosquitoes in the transmission of *Rickettsia* is not yet well established, it has been shown that *An. gambiae* can transmit *Rickettsia felis*, leading to transient rickettsial infections in mice [91]. However, further research is required to determine the role of *Anopheles* as a competent vector, and not just a carrier, taking into account established vector incrimination criteria [92]. Likewise, it is essential to confirm the presence of *Rickettsia* and characterize the species involved and their pathogenicity [90].

### Insights into the Mycobiota of *Anopheles darlingi*

The fungal genera identified as potentially comprising the *An. darlingi* core mycobiota correspond to fungi consistently detected in mosquitoes from all regions, included filamentous fungi such as *Talaromyces*, *Diaporthe*, *Aspergillus*, *Cladosporium*, *Alternaria* and *Penicillium* and the yeasts *Candida* and *Pichia*, among others (Fig. S3d). According to the available literature, there are no prior reports of fungal microbiota characterization using metabarcoding approaches in adult *An. darlingi*; however, a previous study on the larvae reported the presence of *Penicillium* [21]. Studies focusing on other mosquito genera, such as *Aedes* and *Culex*, have revealed similar fungal community compositions, with *Aspergillus*, *Cladosporium*, *Penicillium*, *Trichoderma*, *Candida*, *Cryptococcus* and *Rhodotorula* among the predominant taxa [25, 93]. All these genera were also detected in *An. darlingi* natural populations across the three study regions.

Furthermore, identified fungal OTUs within the genera *Fusarium*, *Metarhizium* and *Beauveria* include entomopathogenic species with potential application in vector control [94, 95]. Specifically, *Metarhizium anisopliae* and *Beauveria bassiana* have demonstrated efficacy in the biological control of *An. gambiae* larvae in Africa [96]. Besides, *M. anisopliae* reduced feeding and reproductive capacity in adult *An. gambiae* mosquitoes [97]. Until now, only *Fusarium* has been reported in *Anopheles* mosquitoes in the Neotropics, specifically in *An. albimanus* larvae and adults from Perú [98]. The findings from this study open new possibilities for exploring the use of entomopathogenic fungi in vector control strategies in the Neotropics.

Mosquitoes acquire their microbiota through various routes, including water from the aquatic habitat during the larval stage, with only a subgroup of microorganisms persisting into adulthood [12]; vertical transmission [99] and sugar or blood feeding [100, 101]. While the acquisition pathways of bacterial microbiota are well documented, less is known about the acquisition of fungal communities. The *An. darlingi* fungal OTUs were predominantly associated with saprotrophic ecological guilds, followed by plant parasites/pathogens, animal parasites/pathogens, and endophytes. It has been suggested that saprophytic and plant-related fungi are acquired by larvae from the breeding sites and are retained in the adult mosquito or are obtained during nectar feeding. In contrast, animal parasitic/pathogenic fungi are more likely to infect mosquitoes through the vector’s cuticle [93, 101]. In this context, the surrounding environment where mosquitoes develop and circulate appears to play a critical role in shaping not only their bacterial microbiota, but also their fungal microbiota, as previously suggested for *Ae. albopictus* [93].

### Geography Shapes the Microbiota of *Anopheles darlingi*

The composition of the microbiota in *An. darlingi* from the Amazonas region differed from that observed in the Bajo Cauca and Pacific regions, possibly influenced by a variety of factors, among them, geographic distance. Alpha and beta diversity analyses revealed a higher diversity of bacterial and fungal microbiota in *An. darlingi* from the Amazonas. In this region, community composition differed significantly from that of the Bajo Cauca and Pacific regions, as evidenced by the Bray-Curtis and unweighted UniFrac distance metrics. A similar pattern in bacterial community structure was previously observed in *An. darlingi* from the Bajo Cauca and Pacific regions [15]. Likewise, the viral component of this vector showed a comparable pattern of regional dissimilarity [16]. Notably, the weighted Unifrac results did not show a consistent degree of dissimilarity between regions, likely because this analysis is more susceptible to changes in abundant lineages [102], which limits its ability to detect differences when low-abundance taxa primarily drive microbial variation.

Using distance decay analysis, we observed weak but statistically significant correlation between microbial community similarity (both bacterial and fungal) and geographic distance in *An. darlingi* populations across the regions. This finding aligns with previous observations that showed a decline in microbiota similarity in *Diabrotica virgifera* (Order Coleoptera) with increasing geographic distance [103], as well as shifts in microbiota composition associated with geographic latitude in *Anastrepha ludens* (Order Diptera) [104]. In addition, various studies have shown that the collection site, considered as a geographic factor, influenced differences in mosquito microbiota composition [15, 105]. Results are consistent with the proposed bacterial taxon-area relationship, which indicates a tendency toward lower microbial community similarity between geographically distant locations, mainly due to environmental heterogeneity [106]. In this study, the *An. darlingi* specimens originated from three different ecoregions with variable resource availability and climatic conditions [28], and host availability and abundance [6]. In addition to environmental filtering, dispersal limitation may also contribute to the observed patterns. The *An. darlingi* collection sites are separated by the Andes mountain range, a major geographical barrier that could restrict mosquito movement [3] and associated microbial dispersal among regions, thereby contributing to the observed differences in mosquito microbiota composition. These factors could partially explain the observed differences in microbiota composition among regions, as previously suggested [13, 107].

In addition to geographical distance, other factors may contribute to the observed differences in *An. darlingi* bacterial and fungal microbiota composition. Previous studies in insects have reported that microbiota composition is associated with host genetic background, as shown in models such as mosquitoes [11], wasps [13] and water fleas [108]. Although genetic difference was not assessed in the present study, earlier research has documented that *An. darlingi* from Colombia is structured into two populations; one located west of the Andes (including the Bajo Cauca and Pacific regions), which is genetically closer to the Central American populations, and the other located to the east (the Amazonas region), which is genetically related to Brazilian populations [3, 109]. The reported genetic structure and the results on its microbial composition allow hypothesizing that the genetic background of *An. darlingi* might be shaping its microbiota. Therefore, future studies are necessary to evaluate the relationship between host genetics and microbial composition in this vector.

### Potential Microbial Interactions and Their Ecological Role in Host Biology

This study aimed to generate hypotheses regarding intra-kingdom interactions between components of the host microbiota. To achieve this, we constructed co-occurrence networks to identify microbial components (ASVs and OTUs) that respond similarly to changes in the host and environment. This approach revealed that mosquitoes from the Amazonas region had the highest number of unique ASVs and OTUs, and their co-occurrence networks exhibited higher degree values, mainly due to the bacterial genera *Asaia*, *Prevotella*, and *Acinetobacter*. Notably, *Asaia* was previously reported as a predominant bacterium in *An. darlingi* [110]; it was also proposed as a genus of interest because it induces the expression of antimicrobial peptides in vitro that may interfere with the *Plasmodium* life cycle [111]. Some of its characteristics, such as genome reduction, loss of mobile elements, and loss of motility, among others, shape *Asaia* as a symbiont in *An. darlingi* [112]. Furthermore, in a metatranscriptomic study, *Asaia* transcripts were linked to carbohydrate and amino acid transport and metabolism, as well as vitamin biosynthesis [63]; these processes may benefit *An. darlingi*. Also, *Asaia*’s role as a keystone taxon in the microbial co-occurrence network further underscores its ecological relevance.

The co-occurrence networks also revealed that some of the most abundant bacterial genera, such as *Cupriavidus*, *Bradyrhizobium* and *Mesorhizobium* were not part of network modules. In the Amazonas region, the bacteria with the highest degree of connectivity were *Klebsiella*, *Asaia* and *Acinetobacter*, which have been previously identified and evaluated in mosquito vectors [75, 113]. In contrast, other genera present within the main modules, such as *Blautia*, *Akkermansia*, *Prevotella* and *Bacteroides*, remain largely unexplored in terms of their interaction with mosquitoes. To date, the only evidence linking these taxa to mosquito-associated microbiota is the reported co-occurrence of *Blautia* and *Asaia* in *An. darlingi* [110]. Of note, inferences about microbial interactions derived from co-occurrence networks are inherently limited. These networks primarily reflect taxa that occupy similar ecological niches instead of direct causal relationships. Nonetheless, these analyses provide a valuable overview of microbial community structure and help to prioritize taxa for future studies aimed at understanding specific ecological or functional interactions [114].

Furthermore, an interesting overall finding from our studies is that mosquitoes from the Amazonas harbored a more abundant and diverse bacterial and fungal community than those from the other two regions. In Colombia, this region accounts for a relatively low proportion of the reported malaria cases, approximately 7.7% of the total, whereas the Pacific and Bajo Cauca regions report 54.5% and 31.5% of cases, respectively [2]; a trend that has remained consistent over time [115]. Similarly, greater bacterial diversity was reported in *Anopheles sinensis* collected from areas with lower malaria transmission [116]. These findings raise the question of whether the mosquito microbiota composition and diversity influence its capacity to transmit the parasite. Future research would benefit from including the analysis of *An. darlingi* populations from areas with lower malaria transmission, where this vector is also present [4], such as the Andean, Caribbean, or Orinoco regions [2].

Finally, although this study provides valuable insights into the microbiota of *An. darlingi* mosquitoes and offers interesting findings, it is important to acknowledge some limitations. These include the sample size and the fact that microbiota was assessed from whole mosquitoes, which prevents conclusions about tissue-specific bacterial and fungal presence. Future studies should consider these factors and the role of determinants, such as host genetic background, in bacterial and fungal composition. Furthermore, it will be relevant to evaluate the transstadial dynamics of fungal communities and the potential of specific fungal strains for vector-control interventions.

## Conclusion

This study advances the understanding of *An. darlingi* bacterial and fungal microbiota in Colombia, revealing differences in microbial composition and diversity across geographical settings. Higher alpha diversity was observed in mosquitoes from the Amazonas, while beta diversity analyses indicated differences in both bacterial and fungal community composition among regions, particularly between Amazonas and the other two regions. In addition, the microbial communities of *An. darlingi* showed decreased similarity with increasing geographic distance in both bacterial and fungal communities. The characterization of the microbiota composition in this vector allowed the identification of taxa shared across all sampled regions, suggesting a core microbiota in Colombian *An. darlingi*. In addition, bacterial taxa associated with *An. darlingi* included the genera *Serratia*, *Enterobacter*, *Delftia*, *Pantoea*, *Pseudomonas* and *Escherichia*; some strains within these genera have been previously reported to exhibit potential inhibitory activity against *Plasmodium* parasites. Similarly, fungal taxa included *Fusarium*, *Metarhizium* and *Beauveria*, contain members with entomopathogenic potential useful for vector control. These findings provide insight into the structure of the microbiota of *An. darlingi* and may serve as the basis for future research aimed at understanding its role in vector control and its influence on vector competence in epidemiologically relevant regions.

## Supplementary Information

Below is the link to the electronic supplementary material.


Supplementary Material 1



Supplementary Material 2


## Data Availability

The raw Illumina sequencing reads generated during this study are available in the NCBI database Sequence Read Archive under BioProject accession number PRJNA1276476 (https://www.ncbi.nlm.nih.gov/sra/PRJNA1276476), BioSamples SAMN49073438 to SAMN49073491.
