# Supplementary material for: Bacterial and Fungal Microbiota in Anopheles darlingi Exhibit Differences in Diversity Across Three Main Colombian Malaria-endemic Regions
Source: Microb Ecol. 2026 Jul 10;89:167. doi: 10.1007/s00248-026-02829-9 (PMC13534409; doi:10.1007/s00248-026-02829-9)
Supplement: Supplementary file 1 [file 248_2026_2829_MOESM1_ESM.docx]

**Bacterial and fungal microbiota in *Anopheles darlingi* exhibit differences in diversity across three main Colombian malaria-endemic regions**

Paola Muñoz-Laiton^1^, Juan C. Hernandez-Valencia^1^, Juan Pablo Isaza^2^, Maisa da Silva Araújo^3^, Joana Falcão Salles^4^, Margarita M. Correa^1^

^1^ Grupo Microbiología Molecular, Escuela de Microbiología, Universidad de Antioquia, Medellín 050010, Colombia

^2^ Grupo Biología de Sistemas, Escuela de Ciencias de La Salud, Facultad de Medicina, Universidad Pontificia

Bolivariana, Medellín 050031, Colombia

^3^ Plataforma de Produção e Infecção de Vetores da Malária (PIVEM), Laboratório de Entomologia, Fiocruz Rondônia,

Porto Velho 76812-245, RO, Brazil
^4^ Groningen Institute for Evolutionary Life Sciences (GELIFES), University of Groningen, Nijenborgh 7, P.O. Box

11103, Groningen 9700 CC, The Netherlands

**Supplementary figures**

**Fig. S1** Rarefaction curves of observed richness per sample in *Anopheles darlingi*……………………………1

**Fig. S2** Taxonomic classification of **(a)** bacterial ASVs and **(b)** fungal OTUs in *Anopheles darlingi* from Colombia at the phylum level…………………………………………………………………………………...2

**Fig. S3** Unique and shared microbiota in *Anopheles darlingi* across regions…………………………………..3

**Fig. S4** Principal Coordinates Analysis (PCoA) of bacterial ASVs in *Anopheles darlingi* based on the weighted UniFrac distance matrix (outlier samples AB6, PB1, PB3 and PB10 excluded)………………………………..4

**Fig. S5** Principal Coordinates Analysis (PCoA) of bacterial ASVs in *Anopheles darlingi*. based on **(a)** Bray-Curtis dissimilarity matrix, **(b)** unweighted UniFrac matrix and **(c)** weighted UniFrac matrix. Each point in the plot represents an individual sample. The percentage of variation explained by each PCoA axis is indicated in parentheses. PERMANOVA statistics are shown at the bottom of each plot……………………………………5

**Fig. S6** Co-occurrence networks of bacterial ASVs and fungal OTUs identified in *Anopheles* *darlingi* from Amazonas, Pacific and Bajo Cauca regions……………………………………………………………………..6

**Fig. S1** Rarefaction curves of observed richness per sample in *Anopheles darlingi*. **(a)** Number of observed bacterial ASVs after rarefaction to 87,517 sequences of the 16S rRNA gene (V3-V4 region), generated using QIIME2 view and **(b)** number of observed fungal OTUs after rarefaction to 105,044 ITS2 sequences. Each line represents an individual sample, based on sequencing data processed in R

**Fig. S2** Taxonomic classification, at the phylum level of **(a)** bacterial ASVs and **(b)** fungal OTUs in *Anopheles darlingi* from Colombia. The figure displays the ten most abundant taxa based on relative abundance; less represented taxa are grouped under the category "Other"

**Fig. S3** Unique and shared microbiota in *Anopheles darlingi* across regions. **(a)** Number of unique and shared bacterial ASVs among regions, **(b)** word cloud displaying the most abundant genera among the 133 bacterial ASVs shared across all regions, **(c)** number of unique and shared fungal OTUs among regions and **(d)** word cloud showing the most abundant genera among the 172 fungal OTUs shared across all regions

**Fig. S4** Principal Coordinates Analysis (PCoA) of bacterial ASVs in *Anopheles darlingi* based on the weighted UniFrac distance matrix (outlier samples AB6, PB1, PB3 and PB10 excluded). Each point in the plot represents a sample. The percentage of variation explained by each PCoA axis is shown in parentheses. PERMANOVA statistics are presented at the bottom of the plot

**Fig. S5** Principal coordinates analysis of bacterial ASVs in *Anopheles darlingi*, based on **(a)** the Bray-Curtis dissimilarity matrix, **(b)** the unweighted UniFrac matrix and **(c)** the weighted UniFrac matrix. Each point in the plot represents an individual sample. The percentage of variation explained by each PCoA axis is indicated in parentheses. PERMANOVA statistics are shown at the bottom of each plot

**Fig. S6** Co-occurrence networks of bacterial ASVs and fungal OTUs identified in *Anopheles* *darlingi* from Amazonas (AM), Pacific (PC) and Bajo Cauca (BC) regions. **(a)** Bacteria and **(b)** fungi. Nodes with the same color correspond to the same region. Only correlations with ρ > 0.7 and *p* < 0.05 were included
